# Characteristics and quality of life of substance users and their caregivers

**DOI:** 10.1097/MD.0000000000029699

**Published:** 2022-08-05

**Authors:** Jadranka M. Maksimovic, Olivera B. Sbutega, Aleksandar D. Pavlovic, Hristina D. Vlajinac, Ivana I. Kavecan, Isidora S. Vujcic, Sandra B. Grujicic Sipetic

**Affiliations:** a Institute of Epidemiology, Faculty of Medicine, University of Belgrade, Belgrade, Serbia; b Special Hospital for Addiction Diseases, Belgrade, Serbia; c Faculty of Medicine, University of Novi Sad, Novi Sad, Serbia; d Department of Pediatrics, Institute for Children and Youth Health Care of Vojvodina, Novi Sad, Serbia.

**Keywords:** addiction diseases, caregivers, depression, quality of life, substance users

## Abstract

The correlation between substance use and depression has been emphasized in the literature. Substance use disorders can also adversely affect the caregivers of drug-addicted persons.

A cross-sectional study was conducted at the Special Hospital for Addiction Diseases in Belgrade in 2015 to analyze the characteristics, consequences, and health-related quality of life of drug users and their caregivers. The sample comprised 136 users of various substances, and 136 caregivers. A questionnaire on socio-demographic characteristics, the Short Form Health Survey 36 (SF-36), and Beck Depression Inventory were administered to all participants.

According to multivariate logistic regression analysis, compared with caregivers, substance users were significantly more frequently male (*P* < .001), ≤ 39 years old (*P* < .001), and more frequently reported the use of sedatives (*P* = .009) and smoking (*P* < .001). Some level of depression was present in all participants, but severe forms were more frequent in substance users (*P* = .010). Among substance users, mean scores of SF-36 domains ranged from 56.62‒87.17, and among their caregivers, from 50.37‒75.07; however, the difference was significant only for the health change domain (*P* = .037), the score for which was lower in caregivers.

Substance users suffered from more severe forms of depression compared to their caregivers, who had lower SF-36 scores in the domain of health change.

## 1. Introduction

The prevalence and severity of addiction disorders are increasing worldwide. In 2018, the United Nations Office on Drugs and Crime reported that there were an estimated 275 million people who abused drugs at least once in 2017, while 31 million suffered from substance use disorders (SUD).^[1]^ According to the 2014 National Survey on Lifestyles of Citizens in Serbia,^[2]^ 72.2% of the adult population consumed alcohol in the last year, and harmful or problematic drinking was reported by 6.2%. The same data show that the prevalence of illicit drug use in the Serbian population aged 18 to 64 years was lower than that in the majority of EU countries, with a lifetime use of 8.0% and 1.7%, respectively, in the last 12 months. The most consumed illegal drug was cannabis, with a lifetime prevalence of 7.7%, and problematic cannabis use in the last 12 months was 0.5%. Daily use of sedatives and hypnotics in the last 30 days was reported by 4.4% of adults.

People suffering from SUD are at increased risks of impaired physical and mental health, shortened life expectancy, and socioeconomic consequences.^[3,4]^ The most noticeable health consequences of SUD are acute life-threatening effects, such as intoxication and overdose; however, SUD is also linked to infectious (HIV, viral hepatitis, etc.) and noncommunicable diseases (cardiovascular diseases, stroke, various cancers, etc.).^[4]^ Similarly, substance use is associated with the leading causes of injury and death during adolescence (road traffic traumatism, suicide), as well as with risky behavior during this period.^[4]^ In addition, healthcare professionals’ prejudices and their stigmatization of substance users can result in insufficient health care.^[5]^The correlation between substance use and mental illness, especially depression, has been emphasized in the literature.^[6–8]^ Rossheim et al^[8]^ concluded that depressive symptoms can remain undiagnosed because they are sometimes perceived as a consequence of life circumstances.

Previous studies have shown that family members have a significant influence on the initiation of treatment for addiction disorder, compliance, and the ultimate outcome of treatment.^[9,10]^ SUD can also adversely affect the family members, partners, and caregivers of drug-addicted persons, thereby resulting in the deterioration of their physical and mental health, social life, and living conditions.^[11–13]^ The *stress-strain-coping-support* (SSCS) model—established to describe the effects of SUD on drug addicts’ family members—has been well documented.^[14]^ The model explains that substance users’ behavior triggers stress among family members, which consequently causes strain (physical and emotional symptoms). The amount of stress and strain is mediated by 2 key factors—the way family members of persons with SUD cope with the problem and the quality of social support they can access. Some interventions for affected family members are based on the SSCS model.^[14]^ Orford^[12]^ summarized the core of affected family members’ experiences and reported that family members of drug users most often experience concern for their relatives, family disharmony, exposure to threats, lack of accurate information, coping with several dilemmas, exhibiting high levels of stress and impaired health, and lacking adequate health care. Marcon et al^[15]^ showed that caregivers have even worse health-related quality of life (HRQoL) than substance users. However, the findings for other diseases are different. For example, Lubomsi et al^[16]^ showed that patients with Parkinson disease have a significantly lower HRQoL than their caregivers, with the largest differences noted in the physical rather than mental limitations. Furthermore, family members of those with SUD often face social stigma.^[17,18]^

Similar to other health-related contexts (e.g., HIV and mental illness), stigma is a powerful social determinant of SUDs and can lead to the development of SUDs among people living with a wide range of stigmatized statuses, as well as undermine the recovery efforts among people with SUDs.^[19]^ Faghih and Pahlavanzadeh^[20]^ and Sakiyama et al^[21]^ stressed the importance of treating caregivers as a vulnerable group and the need to support programs for family members of substance users.

For this purpose, this study aimed to analyze the characteristics, consequences, and health-related quality of life of drug users and their family caregivers.

## 2. Methods

A cross-sectional study was conducted at the Special Hospital for Addiction Diseases in Belgrade in 2015. The hospital offers comprehensive care for people with all types of addiction (alcohol addiction, opiate addiction, addiction to psychostimulants and hallucinogens, and behavioral addiction, including pathological gambling), as well as hospital and outpatient treatment.

### 2.1. The population

Our sample consisted of 136 consecutive patients who were users of various substances, and 136 caregivers with whom they live.

The inclusion criteria for users were patients at the special hospital, with any history of dependence on any substance. Patients with cognitive impairment and those younger than 16 were excluded. The inclusion criteria for caregivers were that they were close family members living with the interviewed user and were responsible for the therapy prescribed to patients.

### 2.2. Data collection

Participation was completely voluntary and anonymous, and all participants provided their consent to participate in the study. Substance users and their caregivers were asked to complete a questionnaire after providing a detailed explanation of the study aim. The main researcher was a trained medical staff member at the special hospital. The Institutional Review Board of the Faculty of Medicine, University of Belgrade, approved this research.

Data were collected from all participants using the following 3 questionnaires:

1. A questionnaire about socio-demographic characteristics (such as age, gender, marital status, educational level, socioeconomic status and if it worsened because of addiction), personal and family history (lifestyle habits, consumption of drugs, health status), and clinical characteristics of addiction.
2. The Short Form Health Survey 36 (SF-36), a multipurpose participant-reported survey of HRQoL, was developed by the RAND Corporation.^[22]^ It comprises 36 questions, divided into 8 subscales measuring the following domains: physical functioning, role limitations due to physical health, limitations due to emotional problems, energy/fatigue, emotional well-being, social functioning, pain, general health, and health change.^[22]^
3. Beck’s Depression Inventory is a 21-question multiple-choice self-report inventory and is one of the most widely used psychometric tests for measuring depression severity.^[23]^ It was developed by Aaron T. Beck, an American psychiatrist.^[23]^

The SF-36 and Beck Depression Inventory were translated to the Serbian language.^[24,25]^

### 2.3. Data analysis

Statistical analysis was performed using the statistical software SPSS Version 17.0 (SPSS Inc., Chicago, Illinois, USA). The categorical variables were presented as counts and percentages, and continuous variables were described as means ± standard deviation (SD). For the data analysis, univariate and multivariate logistic regression analyses were used. All variables that significantly differed (*P* ≤ .10) between the compared groups in univariate analysis were included in the multivariate analysis. A 2-tailed *P* < .05 was considered statistically significant.

### 2.4. Ethical approval

The study received ethical approval from the Ethics Committee of the Faculty of Medicine in Belgrade. All patients provided written informed consent (Approval No. 29/XII-4).

## 3. Results

Table 1 presents the characteristics of the substance users who participated in the survey. Patients of the special hospital who participated in the study most often suffered from heroin addiction, followed by alcohol consumption and other illicit substance addictions. At the time of the study, the majority of users underwent hospital or substitution treatment, whereas a smaller number were in rehabilitation and outpatient treatment (Table 1). Most of the caregivers were parents of substance users, followed by spouses and siblings (Table 2).

**Table 1:** Characteristics of substance users—type of substance used and type of treatment.

	N = 136	%
Substance		
Heroin	68	50.0
Alcohol	26	19.1
Cannabis	9	6.6
Other opioids	6	4.4
Other substances*	27	19.9
Type of treatment		
Hospital	57	41.9
Substitution therapy	47	34.6
Rehabilitation	25	18.4
Outpatient	7	5.1

**Table 2:** Caregivers’ relationships with the substance users.

Caregivers	N = 136	%
Parent	83	61.0
Spouses	19	14.0
Siblings	16	11.8
Partner	5	3.7
Other*	13	9.5

The sociodemographic characteristics and lifestyle habits of the substance users and their caregivers are presented in Table 3. Substance users were most frequently male (86.6%), ≤ 39 years old (64.7%), single (56.6%), from urban areas (94.1%), with secondary school or faculty (86.8%), and with middle socioeconomic status (53.7%), which worsened because of addiction in 66% of the participants. Most substance users were smokers (89.0%), and 36.8% reported the use of sedatives. Caregivers were most frequently female (72.8%), ≥ 50 years old (57.4%), married or living with a partner (70.6%), from urban areas (87.5%), with secondary school or faculty (85.3%), and with middle socioeconomic status (71.3%), which worsened for 53.7% of them. Less than half (41.2%) of the caregivers reported smoking and 29.4% reported the use of sedatives. All these differences between substance users and their caregivers were significant at the level of *P ≤ *.10, with the exception of education level.

**Table 3:** Demographic and socioeconomic characteristics and lifestyle habits of substance users and their caregivers.

Participants’ characteristics	Users (N = 136)	Caregivers (N = 136)	*P* value*
	N (%)	N (%)	
Gender			
Female	18 (13.2)	99 (72.8)	
Male	118 (86.6)	37 (27.2)	< 0.001
Age			
≤ 39	88 (64.7)	26 (19.1)	
40–49	28 (20.6)	32 (23.5)	
≥ 50	20 (14.7)	78 (57.4)	< 0.001
Background			
Urban	128 (94.1)	119 (87.5)	
Rural	8 (5.9)	17 (12.5)	0.065
Marital status			
Single	77 (56.6)	11 (8.1)	
Married	22 (16.2)	77 (56.6)	
Common-law married	16 (11.8)	19 (14.0)	
Divorced	17 (12.5)	12 (8.8)	
Widowed	4 (2.9	17 (12.5)	< 0.001
Education level			
Incomplete elementary	2 (1.5)	7 (5.1)	
Elementary school	16 (11.8)	13 (9.6)	
Secondary school	87 (64.0)	82 (60.3)	
Faculty	31 (22.8)	34 (25.0)	0.725
Self-reported socioeconomic status			
Low	44 (32.4)	23 (16.9)	
Middle	73 (53.7)	97 (71.3)	
High	19 (14.0)	16 (11.8)	0.071
Addiction worsened socioeconomic status			
Yes	90 (66.2)	73 (53.7)	
No	46 (33.8)	63 (46.3)	0.036 Consumption of sedatives
Yes	50 (36.8)	40 (29.4)	
No	86 (63.2)	96 (70.6)	<0.001
Smoking status			
Smoker	121 (89.0)	56 (41.2)	
nonsmoker	15 (11.0)	79 (58.1)	<0.001

According to the Beck Depression Inventory (Table 4), although some level of depression was present in all participants, more severe forms of depression were more frequent in substance users than in their caregivers (*P* = .002). None of the study participants had depression in their personal history.

**Table 4:** Beck Depression Inventory scores of the substance users and their caregivers.

Severity of depression	Users (N = 136)	Caregivers (N = 136)	*P* value *
	N (%)	N (%)	
Minimal depression	81 (59.6)	103 (75.7)	
Mild depression	20 (14.7)	16 (11.8)	0.002
Moderate depression	20 (14.7)	12 (8.8)	
Severe depression	15 (11.0)	5 (3.7)	

Table 5 displays the differences between substance addicts and caregivers in terms of the mean SF-36 scores measuring HRQoL. In the substance users, mean scores of SF-36 domains ranged from 56.62 to 64.04, with the exception of the physical functioning, pain, and health change domains, for which they were higher. The scores of SF-36 domains in caregivers were similar to those of the substance users; however, 3 of them were significantly lower, namely, scores for physical functioning (*P* < .001), pain (*P* = .003) and health change (*P* < .001).

**Table 5:** SF-36 scores of the substance users and their caregivers.

SF-36 domain	Users (N = 136)	Caregivers (N = 136)	*P* value*
	(Mean ± SD)	(Mean ± SD)	
Physical functioning	87.17 ± 19.63	75.07 ± 25.20	< 0.001
Role limitations due to physical health	63.05 ± 41.27	69.67 ± 40.87	0.185
Role limitations due to emotional problems	56.62 ± 44.32	64.21 ± 40.90	0.143
Energy/fatigue	56.58 ± 22.52	58.12 ± 21.84	0.565
Emotional well-being	58.26 ± 23.01	60.38 ± 20.29	0.420
Social functioning	62.78 ± 31.49	66.91 ± 26.55	0.242
Pain	76.54 ± 28.21	66.54 ± 25.67	0.003
General health	64.04 ± 19.94	61.62 ± 17.34	0.284
Health change	65.07 ± 31.37	50.37 ± 22.56	< 0.001

According to the multivariate analysis, compared to the caregivers, substance users were significantly more frequently male (*P* < .001), ≤ 39 years old (*P* < .001), and most frequently reported the use of sedatives (*P* = .009), followed by smoking (*P* < .001). They also more frequently had severe forms of depression (*P* = .010). The SF-36 score of the health change domain was significantly lower in the caregivers (*P* = .037).

## 4. Discussion

The results of our study showed that parents comprised the majority of caregivers of those with SUD. A study conducted in Brazil on family members affected by their relative substance abuse found that parents were the largest group.^[21]^ Orford^[12]^ described that partners and parents are most frequently affected by family members’ drug addiction. Moreover, most of the caregivers of Australian patients suffering from psychosis were also parents.^[26]^ Parents are the most common caregivers of patients with developmental disabilities.^[27]^ The high representation of parents among caregivers in this study had an impact on the distribution of some of their characteristics—caregivers were more likely to be older, officially married, or common-law partnered. A similar study conducted by Marcon et al^[15]^ also found that caregivers are, on average, older and more likely to be living with a partner.

According to our results, a gender difference was observed between substance users and their caregivers. As Marcon et al^[15]^ described, women are more likely to take on the role of caregivers, whereas men tend to consume illicit drugs more frequently. In the United States, women make up most caregivers in general, as well as for people suffering from dementia.^[28]^ A study on caregivers conducted in Spain found that women were more likely than men to have health problems and work or financial problems.^[29]^

As expected, most of our substance users were smokers. The association between cigarette consumption and various patterns of substance use is well known, and scholars often describe tobacco use as a gateway to substance consumption.^[30–32]^ The correlation between smoking and substance use can be explained by external factors that simultaneously influence behavior, as well as by genetic factors.^[32]^

The worsening of socioeconomic status in our substance users was expected and understandable. Although caregivers in this study were less likely to report that their socioeconomic status had worsened, it is important to emphasize that more than half of them experienced socioeconomic deterioration due to SUD. Lai^[33]^ explained that providing care to a family member significantly creates a burden and financial consequences.

Consistent with the results of Marcon et al,^[15]^ our study found that compared to the caregivers, the substance users had significantly lower scores on the Beck Depression Inventory.^[15]^ Moreover, it has been reported that substance use is associated with mental illness, especially anxiety and depression.^[4,6–8]^

Our study found that caregivers had lower values in several domains of HRQoL compared to substance users; these domains included physical functioning, pain, and health change. However, the association was significant independently of other factors only for the health change domain, and values for other domains were similar in both groups. This is similar to the findings of Marcon et al,^[15]^ who reported that caregivers had lower values in 4 similar domains: physical functioning, role limitations due to physical health, pain, and energy/fatigue. In their scoping review, Birkeland et al^[13]^ emphasized that substance use has a great impact on the quality of life of partners of people with SUD. On the contrary, Lin et al^[9]^ highlighted that family support positively affects the quality of life of substance users. Caregivers of patients suffering from schizophrenia, dementia, stroke, and cancer may also be at risk of deteriorating health status; meanwhile, studies on schizophrenia and stroke have also reported a deterioration in the quality of life of caregivers.^[34–37]^ Carra et al’s^[38]^ study showed a link between the highly expressed emotions of family members, who are most often caregivers of people with schizophrenia, thus indicating a need to improve support for caregivers through psychoeducational interventions. In a study of 131 caregivers of patients suffering from multiple sclerosis, Petrikis et al^[39]^ found that the high depression rates in caregivers were positively correlated with caregiver stress and negatively associated with physical and mental health status, as indicated by SF-36 scores.

In the nationally representative surveys of community-dwelling older adults and their family caregivers residing in the US, Rifin et al^[40]^concluded that caregiver burden is determined more by the caregivers’ characteristics and provision of caregiving tasks than by the characteristics of the care recipient. In a study by Du et al^[41]^ that observed family caregivers of disabled older adults, subjective caregiver burden was negatively associated with all 8 subscales of the SF-36. A study in South Korea found that caregiving has significant adverse effects on caregivers’ multiple dimensions of health.^[42]^ Furthermore, they postulated that the role of caregivers is chosen by those who can give the most time, which can be linked to previous health and socioeconomic disadvantages.^[42]^ Namkung et al^[38]^ emphasized that caregivers reported poorer well-being compared with noncaregivers, and that there is a difference according to the relationship with the person they are caring for. Specifically, parents and spouse caregivers were more affected than sibling caregivers.^[43]^ Farina et al^[28]^ concluded that spouses have a worse quality of life than their care recipients. Additionally, Orford^[12]^ explained that women have more difficulty coping with their relative substance use problems.

Given that we only included patients from the special hospital, one of the limitations of our study could be a sampling bias. Consequently, the sample may not be representative of community-dwelling substance users. We can assume that patients with severe forms of the disease are overrepresented at the hospital, and that there is a lower proportion of patients not living in Belgrade and other major cities, which may be related to a different pattern of substance use. Data were collected through self-reporting, including statements about sensitive personal data, which can lead to recall bias. Conducting a cross-sectional study, we were limited to making temporal and causal inferences.

In this study, the substance users were most frequently single young men (≤ 39 years old), with most of them being smokers, and caregivers were most frequently women aged ≥ 50 years who lived with a partner. Both users and caregivers were mostly from urban areas, with high school or higher education and middle socioeconomic status; moreover, addiction was followed by worsening of socioeconomic conditions, especially among users. Substance users suffered from more severe forms of depression, but their caregivers had a lower HRQoL in the domain of health change. These results indicate the importance of future studies on this topic, as well as the importance of better understanding the consequences of addiction on the quality of life of users and their family members, partners, and relatives. Based on these results, it is recommended to develop a strategy for implementing caregiver support programs.

## Acknowledgments

We deeply appreciate the patients and their caregivers for participating in this study. This work was supported by the Ministry of Education, Science and Technological Development of the Republic of Serbia (grants No. 175042).

## Author contributions

Collection the data: OS

Analyzed the data: AP, IV, IK

Wrote the paper: JM, OS, SGS

Supervision: JM, HV

## Footnotes

* Hallucinogenic drugs, hypnotics, sedatives.

* Friend, colleague, grandmother, grandfather.

* According to univariate logistic regression analysis.

* According to univariate logistic regression analysis.

* According to univariate logistic regression analysis.

## References

[1] World Drug Report 2018. Vienna; 2018. (United Nations publication, Sales No. E.18.XI.9). Available at: https://www.unodc.org/wdr2018/prelaunch/WDR18_Booklet_1_EXSUM.pdf [access date March 18, 2020].

[2] Kilibarda B Mravcik V Sieroslawski J. National survey on life styles of citizens in Serbia 2014 – key findings on substance use and gambling. Belgrade; 2014. Available at: https://www.emcdda.europa.eu/system/files/attachments/11658/GPS-Serbia.pdf [access date January 9, 2018].

[3] Whiteford HA Ferrari AJ Degenhardt L. The global burden of mental, neurological and substance use disorders: an analysis from the Global Burden of Disease Study 2010. PLoS One. 2015;10:e0116820. 25658103 10.1371/journal.pone.0116820 PMC4320057

[4] Schulte MT Hser YI. Substance use and associated health conditions throughout the lifespan. Public Health Rev. 2014;35. Available at: https://web-beta.archive.org/web/20150206061220/http://www.publichealthreviews.eu/upload/pdf_files/14/00_Schulte_Hser.pdf. 10.1007/BF03391702 PMC5373082 28366975

[5] van Boekel LC Brouwers EP van Weeghel J. Stigma among health professionals towards patients with substance use disorders and its consequences for healthcare delivery: systematic review. Drug Alcohol Depend. 2013;131:23–35. 23490450 10.1016/j.drugalcdep.2013.02.018

[6] Wu LT Blazer DG. Substance use disorders and psychiatric comorbidity in mid and later life: a review. Int J Epidemiol. 2014;43:304–17. 24163278 10.1093/ije/dyt173 PMC3997371

[7] Vigo D Jones L Thornicroft G. Burden of mental, neurological, substance use disorders and self-harm in North America: A Comparative Epidemiology of Canada, Mexico, and the United States. Can J Psychiatry. 2020;65:87–98. 31747307 10.1177/0706743719890169 PMC6997975

[8] Rossheim ME Livingston MD Lerch JA. Serious mental illness and negative substance use consequences among adults on probation. Health Justice. 2018;6:6. 29569076 10.1186/s40352-018-0064-7 PMC5864578

[9] Lin C Wu Z Detels R. Family support, quality of life and concurrent substance use among methadone maintenance therapy clients in China. Public Health. 2011;125:269–74. 21414646 10.1016/j.puhe.2011.01.009 PMC3112464

[10] Cornelius T Earnshaw VA Menino D. Treatment motivation among caregivers and adolescents with substance use disorders. J Subst Abuse Treat. 2017;75:10–6. 28237049 10.1016/j.jsat.2017.01.003 PMC5330196

[11] Di Sarno M De Candia V Rancati F. Mental and physical health in family members of substance users: A scoping review. Drug Alcohol Depend. 2021;219:108439. 33333362 10.1016/j.drugalcdep.2020.108439

[12] Orford J. How does the common core to the harm experienced by affected family members vary by relationship, social and cultural factors? Drugs Educ Prev Policy. 2017;24:9–16.

[13] Birkeland B Foster K Selbekk AS. The quality of life when a partner has substance use problems: a scoping review. Health Qual Life Outcomes. 2018;16:219. 30453992 10.1186/s12955-018-1042-4 PMC6245914

[14] Velleman R Orford J Templeton L. 12-Month follow-up after brief interventions in primary care for family members affected by the substance misuse problem of a close relative. Addict Res Theory. 2011;19:362–74.

[15] Marcon SR Rubira EA Espinosa MM. Quality of life and depressive symptoms among caregivers and drug dependent people. Rev Lat Am Enfermagem. 2012;20:167–74. 22481735 10.1590/s0104-11692012000100022

[16] Lubomski M Davis RL Sue CM. Health-related quality of life for Parkinson’s disease patients and their caregivers. J Mov Disord. 2021;14:42–52. 33423435 10.14802/jmd.20079 PMC7840244

[17] McCann TV Lubman DI. Stigma experience of families supporting an adult member with substance misuse. Int J Ment Health Nurs. 2018;27:693–701. 28593690 10.1111/inm.12355

[18] Rafiq M Sadiq R. Caregiver stress, perceived stigma and mental health in female family members of drug addicts: Correlational Study. J Pak Med Assoc. 2019;69:1300–3. 31511715

[19] Earnshaw VA. Stigma and substance use disorders: A clinical, research, and advocacy agenda. Am Psychol. 2020;75:1300–11. 33382299 10.1037/amp0000744 PMC8168446

[20] Faghih M Pahlavanzadeh S. The effect of cognitive behavioral therapy on the burden in drug dependent persons’ caregivers: a randomized controlled clinical trial. Iran J Nurs Midwifery Res. 2019;24:131–6. 30820225 10.4103/ijnmr.IJNMR_86_18 PMC6390435

[21] Sakiyama HM de Fatima Rato Padin M Canfield M. Family members affected by a relative’s substance misuse looking for social support: who are they? Drug Alcohol Depend. 2015;147:276–9. 25541243 10.1016/j.drugalcdep.2014.11.030

[22] Ware JE Jr Sherbourne CD. The MOS 36-item short-form health survey (SF-36). I. Conceptual framework and item selection. Med Care. 1992;30:473–83. 1593914

[23] Beck AT Steer RA Brown GK. Manual for the Beck depression inventory-II. San Antonio, TX: Psychological Corporation, 1996.

[24] Lyon. ProQolid Patient-Reported Outcome and Quality of Life Instruments Database SF-36 Health Survey Serbian version. Available at: http://www.proqolid.org/. 10.1186/1477-7525-3-12 PMC555954 15755325

[25] Novovic Z Mihic L Tovilovic S. Psychometric characteristics of the Beck depression inventory on a Serbian student sample. Psihologija. 2011;44:225–43.

[26] Poon AWC Harvey C Mackinnon A. A longitudinal population-based study of carers of people with psychosis. Epidemiol Psychiatr Sci. 2017;26:265–75. 26847994 10.1017/S2045796015001195 PMC6998635

[27] Wong JD Shobo Y. Types of family caregiving and daily experiences in midlife and late adulthood: the moderating influences of marital status and age. Res Aging. 2017;39:719–40. 28566011 10.1177/0164027516681050

[28] Farina N Page TE Daley S. Factors associated with the quality of life of family carers of people with dementia: A systematic review. Alzheimers Dement. 2017;13:572–81. 28167069 10.1016/j.jalz.2016.12.010

[29] Peña-Longobardo LM Río-Lozano MD Oliva-Moreno J. García-Calvente MDM. Health, Work, and Social Problems in Spanish Informal Caregivers: Does Gender Matter? (The CUIDAR-SE Study). Int J Environ Res Public Health. 2021;18:7332. 34299782 10.3390/ijerph18147332 PMC8306791

[30] Rajabi A Dehghani M Shojaei A. Association between tobacco smoking and opioid use: A meta-analysis. Addict Behav. 2019;92:225–35. 30685521 10.1016/j.addbeh.2018.11.043

[31] Badiani A Boden JM De Pirro S. Tobacco smoking and cannabis use in a longitudinal birth cohort: evidence of reciprocal causal relationships. Drug Alcohol Depend. 2015;150:69–76. 25759089 10.1016/j.drugalcdep.2015.02.015

[32] Cross SJ Lotfipour S Leslie FM. Mechanisms and genetic factors underlying co-use of nicotine and alcohol or other drugs of abuse. Am J Drug Alcohol Abuse. 2017;43:171–85. 27532746 10.1080/00952990.2016.1209512 PMC5493323

[33] Lai DWL. Effect of financial costs on caregiving burden of family caregivers of older adults. SAGE Open. 2012;2:1–14.

[34] McCann TV Lubman DI Boardman G. Affected family members’ experience of, and coping with, aggression and violence within the context of problematic substance use: a qualitative study. BMC Psychiatry. 2017;17:209. 28578666 10.1186/s12888-017-1374-3 PMC5457726

[35] Gavaldà-Espelta E Del Mar Lleixà-Fortuño M Baucells-Lluis J. Effectiveness of the integrated care model Salut+Social in patients with chronic conditions: A mixed methods study protocol. Medicine (Baltim). 2020;99:e19994. 10.1097/MD.0000000000019994 PMC7220253 32384454

[36] Kent EE Rowland JH Northouse L. Caring for caregivers and patients: Research and clinical priorities for informal cancer caregiving. Cancer. 2016;122:1987–95. 26991807 10.1002/cncr.29939 PMC5597246

[37] Opoku-Boateng YN Kretchy IA Aryeetey GC. Economic cost and quality of life of family caregivers of schizophrenic patients attending psychiatric hospitals in Ghana. BMC Health Serv Res. 2017;17(Suppl 2):697. 29219074 10.1186/s12913-017-2642-0 PMC5773918

[38] Carrà G Cazzullo CL Clerici M. The association between expressed emotion, illness severity and subjective burden of care in relatives of patients with schizophrenia. Findings from an Italian population. BMC Psychiatry. 2012;12:140. 22974195 10.1186/1471-244X-12-140 PMC3549782

[39] Petrikis P Baldouma A Katsanos AH. Quality of life and emotional strain in caregivers of patients with multiple sclerosis. J Clin Neurol. 2019;15:77–83. 30618220 10.3988/jcn.2019.15.1.77 PMC6325374

[40] Riffin C Van Ness PH Wolff JL. Multifactorial examination of caregiver burden in a national sample of family and unpaid caregivers. J Am Geriatr Soc. 2019;67:277–83. 30452088 10.1111/jgs.15664 PMC6367031

[41] Du J Shao S Jin GH. Factors associated with health-related quality of life among family caregivers of disabled older adults: a cross-sectional study from Beijing. Medicine (Baltim). 2017;96:e8489. 10.1097/MD.0000000000008489 PMC5682827 29095308

[42] Do YK Norton EC Stearns SC. Informal care and caregiver’s health. Health Econ. 2015;24:224–37. 24753386 10.1002/hec.3012 PMC4201633

[43] Namkung EH Greenberg JS Mailick MR. Well-being of sibling caregivers: effects of kinship relationship and race. Gerontologist. 2017;57:626–36. 26884066 10.1093/geront/gnw008 PMC5881720

